# The actual electronic band structure of a rubrene single crystal

**DOI:** 10.1038/s41598-019-46080-4

**Published:** 2019-07-04

**Authors:** Jun Nitta, Kazumoto Miwa, Naoki Komiya, Emilia Annese, Jun Fujii, Shimpei Ono, Kazuyuki Sakamoto

**Affiliations:** 1 Department of Nanomaterials Science, Chiba University, Chiba, 263-8522 Japan; 2 Central Research Institute of Electric Power Industry, Yokosuka, 240-0196 Japan; 3 Elettra-Sincrotrone Trieste S.C.p.A, I-34149 Trieste, Italy; 4 Istituto Officina del Materiali (IOM)-CNR, Laboratorio TASC, I-34149 Trieste, Italy; 5 Department of Materials Science, Chiba University, Chiba, 263-8522 Japan; 6 Molecular Chirality Research Center, Chiba University, Chiba, 263-8522 Japan; 7 Department of Material and Life Science, Osaka University, Osaka, 565-0871 Japan; 8 Present Address: Centro Brasileiro de Pesquisas Físicas (CBPF), Rua Dr. Xavier Sigaud 150, Urca, Rio de Janeiro, 22290-180 Brazil

**Keywords:** Chemical physics, Organic molecules in materials science

## Abstract

A proper understanding on the charge mobility in organic materials is one of the key factors to realize highly functionalized organic semiconductor devices. So far, however, although a number of studies have proposed the carrier transport mechanism of rubrene single crystal to be band-like, there are disagreements between the results reported in these papers. Here, we show that the actual dispersion widths of the electronic bands formed by the highest occupied molecular orbital are much smaller than those reported in the literature, and that the disagreements originate from the diffraction effect of photoelectron and the vibrations of molecules. The present result indicates that the electronic bands would not be the main channel for hole mobility in case of rubrene single crystal and the necessity to consider a more complex picture like molecular vibrations mediated carrier transport. These findings open an avenue for a thorough insight on how to realize organic semiconductor devices with high carrier mobility.

## Introduction

**Figure 1:**
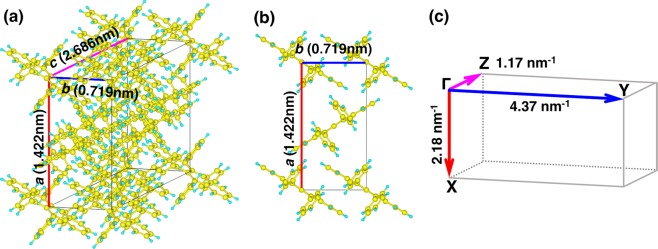
Structure of a rubrene SC. (**a**) Schematic illustration of the molecular arrangement and the unit cell of rubrene SC. (**b**) The *a-b* plane of the rubrene SC that is parallel to the surface (the *c*-axis is normal to the surface). (**c**) The Brillouin zone of a rubrene SC. Γ-Z corresponds to the *c*-axis in real space, and Γ -Y and Γ -X to the *a*- and *b*-axes, respectively.

The state-of-the-art organic-technology, organic light emitting diode (OLED)^1–4^, demonstrates the availability of organic molecules as materials for fascinating printable and flexible electronic devices^5^. However, although a number of powerful organic devices such as organic field-effective transistor^6,7^ and organic solar cell^8,9^ have been proposed, OLED is the only one distributed on the market so far. One of the most crucial problems for realizing the other organic devices is the low charge carrier mobility in most organic crystals. In order to overcome this problem and to fulfill various organic devices, it is indispensable to have a proper understanding on the carrier transport mechanism in organic crystals^10–18^, and therefore a lot of endeavor has been devoted to determine the electronic band structures of organic “single” crystals (SCs)^19–30^. (SCs, in which molecules are highly ordered, would show high carrier mobility due to the absence of defects such as grain boundary that induce disordering of molecules in crystals and scattering of carriers, and thus the reduction of mobility.) Among the organic SCs, rubrene (5,6,11,12-tetraphenyltetracene) SC (Fig. 1) is the one whose electronic band structure was most intensively studied^19–25^ due its high hole mobility as reported by transport measurement (40–45 cm^2^/Vs)^31,32^. Nevertheless, despite of the great experimental and theoretical efforts, there is still no complete consensus between the band dispersion and hole mobility, and not even between the experimentally and theoretical dispersions. This means that the carrier transport mechanism in rubrene SC, which is an essential information for designing organic devices with high carrier mobility, is still an unresolved issue.

Theoretical studies^23–25^ reported the presence of two highest occupied molecular orbital (HOMO)-derived bands with strong anisotropy. All these theoretical studies show the same dispersion behaviors, e.g., the downward dispersions from Γ to Y of the two bands and the degeneration of the two bands at Y, with small variations in the dispersion width from 420 meV^23,25^ to 520 meV^24^ and in the effective hole mass values from *m*_h_*/*m*_e_ = 0.65^25^ to 1.3^23^ in the high mobility direction, the Γ-Y direction in reciprocal space (or the b direction in real space). In contrast to the similar theoretical band structures, the experimentally obtained ones are completely different. One angle-resolved photoelectron spectroscopy (ARPES) measurement carried out using light with photon energy (*hν*) of 21.2 eV^19^ shows two HOMO bands, the same number as that predicted theoretically, but the dispersion width and behavior are different from the theoretical ones. The width is approximately 250 meV, and the two bands do not degenerate at Y. In other ARPES measurements^20–22^ performed with *hν* = 21.2, 30 and 40 eV, only one HOMO-derived band was observed, i.e., even the number of HOMO-derived bands is different from that reported in theoretical studies. Note that despite the discrepant results, all these former studies suggest that the band conductivity is the dominant mechanism of hole transport in rubrene SC. However, the highest hole mobility estimated by ARPES (29 cm^2^/Vs^20^), which should be the value of an ideal rubrene SC if carriers are 100% transport through the bands, is smaller than that obtained by transport measurement where SC with defects and disorder that may lower the mobility from the ideal value was used. Here, we show the HOMO band dispersion of a high-quality rubrene SC obtained using high-resolution ARPES, explain the origin of the contradicted former results by taking the photoelectron diffraction (PED) effect into account, and also discuss the mechanism of carrier transport in rubrene SC.

## Results

### Quality of rubrene SC

**Figure 2:**
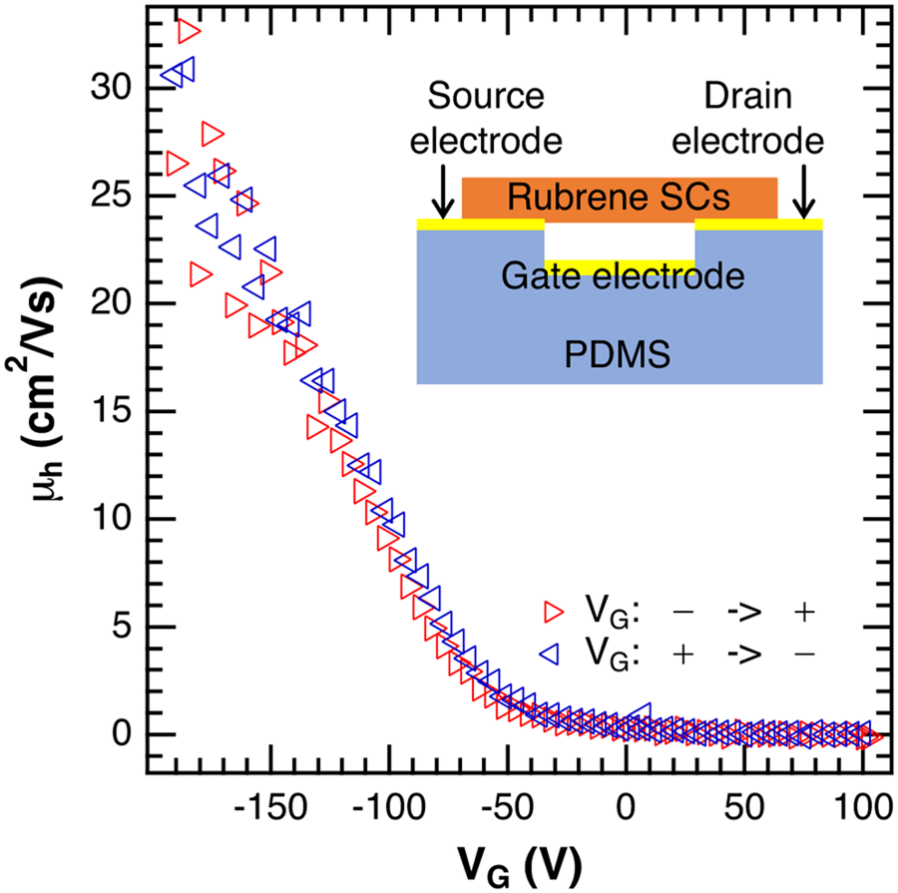
The hole mobility as a function of gate voltage of a FET made by the rubrene SC used in the present study. Red triangles are the hole mobility when changing the gate voltage from negative value to positive value, and blues ones show the hole mobility when changing the gate voltage in the opposite direction. Inset shows the schematic image of the air-gap FET used for measurement.

To confirm the quality of the rubrene SC used in the present study, we made a rubrene SC field-effect transistor (FET) and measured its carrier transport characteristic, i.e., the drain current as a function of gate voltage. The hole mobility was μ_h_ > 30 cm^2^/Vs (Fig. 2) with an average value of 20 cm^2^/Vs, which is comparable to the highest mobility measured with the same method^31,32^. Both the hole mobility and the negligible hysteresis indicating the high stability of the FET device confirm the high quality of the rubrene SC used in the present study.

### Photo-induced damage

**Figure 3:**
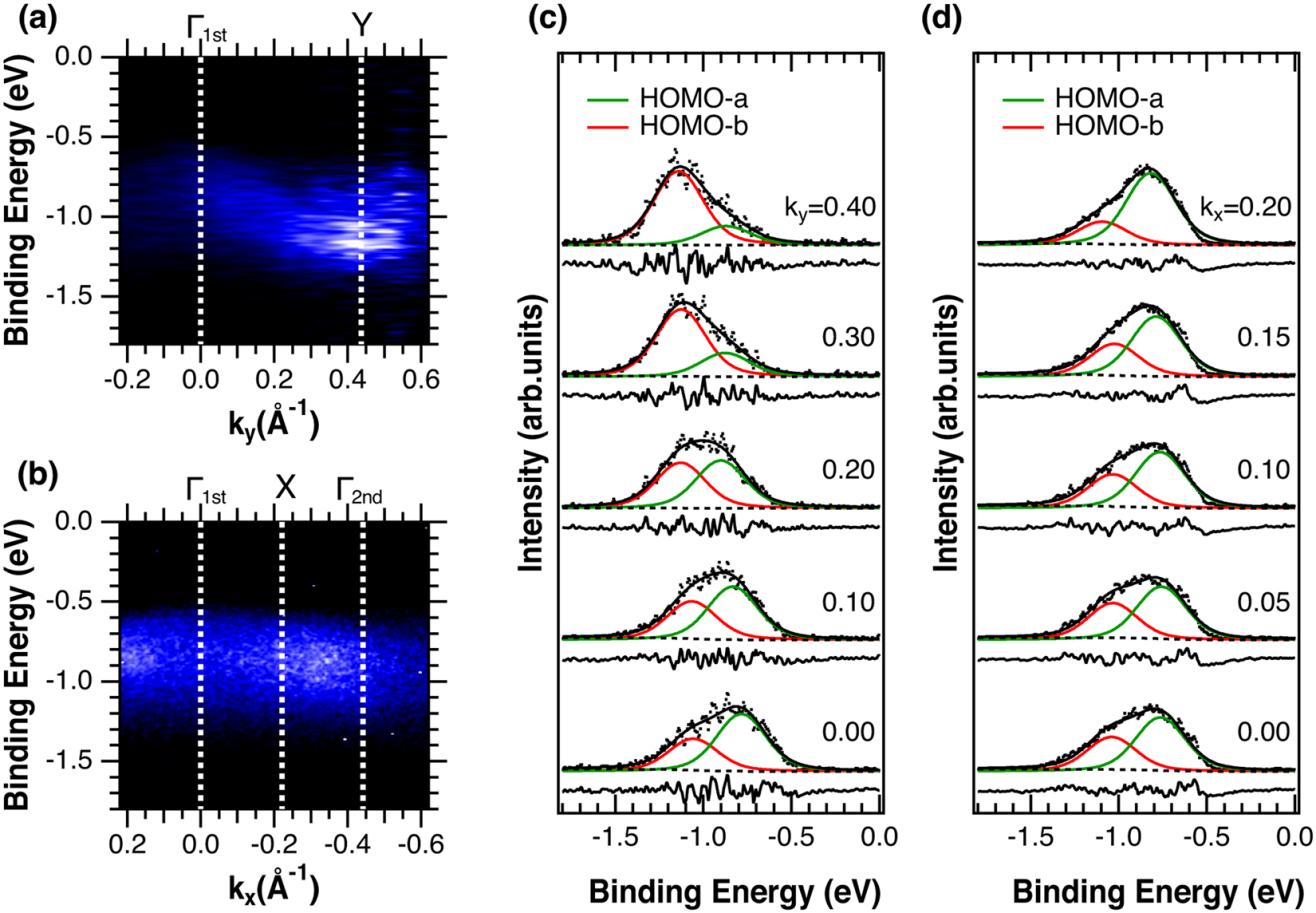
ARPES images and spectra of a rubrene SC obtained with *hν* = 30 eV. (**a**,**b)** Are the images along Γ-Y and Γ-X of the BZ. The brighter area corresponds to higher photoelectron intensity. The horizontal and vertical axes are the wave vector and the binding energy, respectively. The symmetry points of the BZ are denoted by dashed lines. (**c**,**d)** Are the ARPES spectra extracted from (**a**,**b)**. The dots are the experimental data, and the solid lines overlapping the dots are the fitting results obtained by the two components (HOMO-a and HOMO-b) shown below each spectrum. The solid line at the bottom of each spectrum is the residue between the experimental data and the fitting result.

Figure 3a,b shows the ARPES spectral images of the HOMO region of a rubrene SC measured with a SR light of *hν* = 30 eV along the Γ-Y and Γ-X directions of the BZ, respectively. One bright broad band showing a downward dispersion of approximately 350 meV from Γ to Y is observed in Fig. 3a, and one that hardly disperses is observed in Fig. 3b (brighter area corresponds to higher photoelectron intensity). We here notice that no visible light and/or continuous wave laser was irradiated on the sample during the ARPES measurements in the present study, though these lights were believed to be necessary to compensate the charging effect that occurs by the photoemission process and thus to reduce the photo-induced damage of rubrene molecules^19–22^. The negligible photo-induced charging-effect and/or photo-induced damage without irradiating visible light and/or continuous wave laser was also confirmed by the negligible change in the spectral shape and the low background intensity at both sides of the HOMO-derived peak shown in Fig. 3c,d, which are ARPES spectra extracted from Fig. 3a,b, respectively. The HOMO-derived peak shows a significant decrease in intensity with a shift to higher binding energy (*E*_B_) and an increase in the background intensity at the high *E*_B_ side of the HOMO-derived peak in sample damaged by photo-irradiation^20^ (spectra obtained by measuring a lower quality sample, and showing photo-induced damage are shown in Supplementary Fig. S1). Since PES measurements on an amorphous Si, whose carrier mobility is lower than that of rubrene SC^33^, hardly charge up the sample, and because the presence of defect and/or domain boundary would be the origin of charging, the unnecessity of having extra light also supports the high quality of the rubrene SC used in the present study.

### Electronic structure of the HOMO-derived bands

The ARPES spectral images of the HOMO region observed along the Γ-Y and Γ-X directions at *hν* = 40 eV (Supplementary Fig. S2a,b) are identical to those obtained at *hν* = 30 eV. This result indicates the dispersion along the Γ-Z direction of the BZ (the *c*-axis of the crystal in real space) to be negligible, which is consistent with the theoretically predictions^23,24^ and a former experimental result^22^, means that the band dispersion of the HOMO-derived band of rubrene SC shows a rather two-dimensional behavior.

The ARPES spectrum taken at the Γ point (k_//_ = 0 Å^−1^) shows a peak at around *E*_B_ = −0.8 eV together with a clear shoulder structure at around *E*_B_ = −1.1 eV, which indicates the existence of a second component, in both Fig. 3c,d. The existence of two HOMO-derived components is consistent with the presence of two rubrene molecules in the unit cell, and with the number of HOMO-derived bands predicted in all theoretical studies^23–25^ and one experimental study^19^. The intensity of the shoulder increases as k_//_ deviates from 0 Å^−1^ together with a decrease in intensity of the main peak in the Γ-Y direction, while the intensity of the shoulder remains smaller than that of the main peak in the Γ-X direction. In order to obtain quantitative information about the components, the spectra were analyzed by a standard least-square-fitting method using Voigt functions. The solid lines overlapping the dots in Fig. 3c,d are the fitting results obtained using the two components (HOMO-a and HOMO-b) shown below each spectrum. The solid line at the bottom of each spectrum is the difference between the experimental data and fitting result.

## Discussion

**Figure 4:**
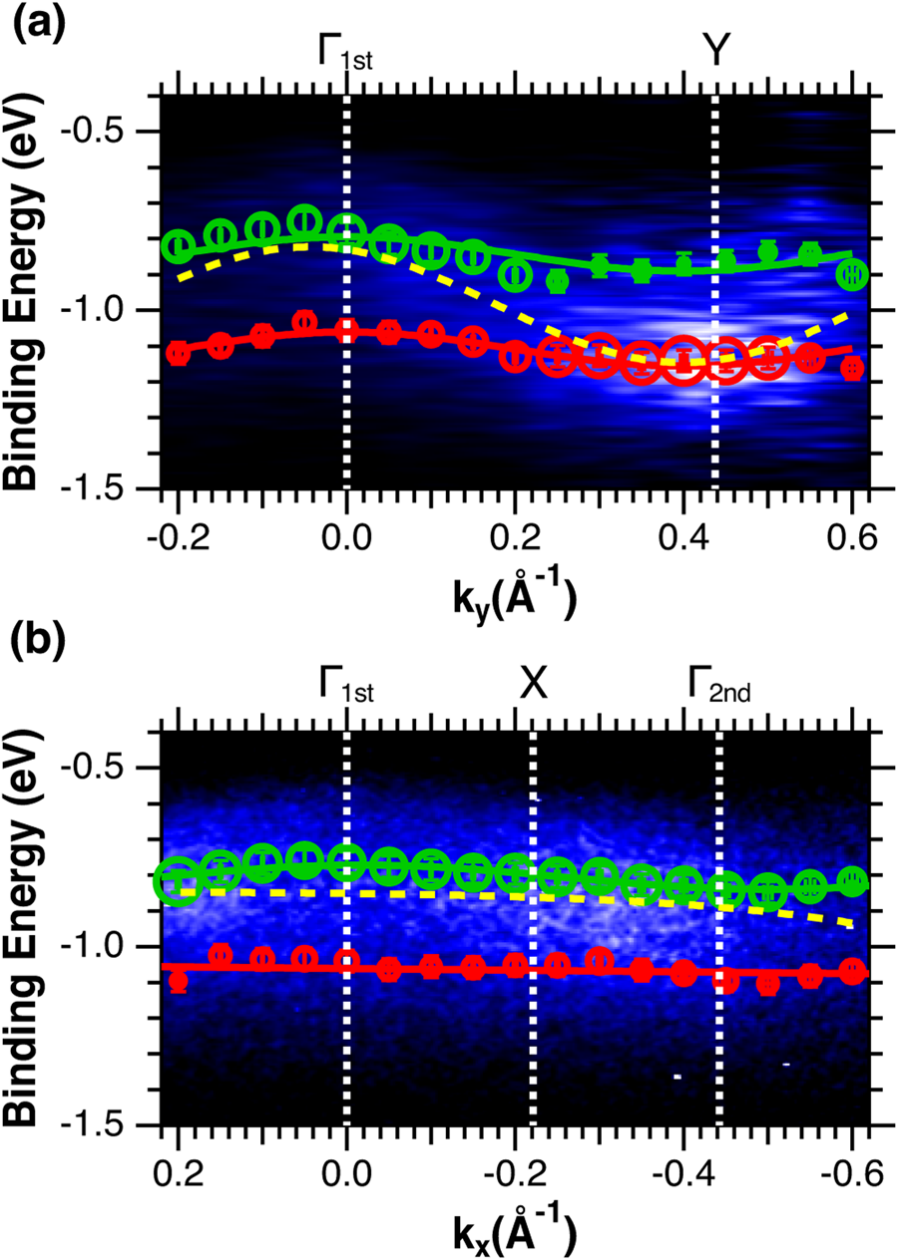
Dispersions of the two HOMO-a and HOMO-b bands obtained from the fitting results. The dispersion along Γ-Y is shown in **a** and that along Γ-X in (**b**). The circles are the two HOMO-derived bands obtained from the fitting shown in Fig. 3. The size of the circles corresponds to the intensity of the components and the experimental error is indicated by bars. The solid lines overlapping the two HOMO bands are the fitting results obtained by using a simple one-dimensional tight-binding model, and the dashed lines are obtained by following the data point with maximum intensity.

In order to understand the carrier transport mechanism in rubrene SC, we first discuss the origin of the disagreement between the present and former experimental studies. Figure 4 shows the dispersion of the two HOMO-derived components obtained from the fitting result superimposed on the ARPES spectral images. The dispersion widths of the two bands obtained by using a simple one-dimensional tight-binding model (solid lines overlapping the open circles), i.e., by least-squares fittings using cosine curves (*E*_B_ = *E*_c_ − 2*t*cos(*bk*_//_)), where *E*_c_, *t*, and *b* are the energy of the band center, the transfer integral and lattice constant of the corresponding crystal axis, respectively, are less than 100 meV along the Γ-Y direction and less than 30 meV along the Γ-X direction. Of these widths, the one obtained along the Γ-Y direction is much smaller than those reported both experimentally^19–22^ and theoretically^23–25^ in former studies. From experimental point of view, there are two reasons that can explain the mismatch, i.e., the difference in the number of HOMO band and the photo-induced damage resulting from the lower sample quality. The later manifests as contribution in the spectral background as explained above. Taking into account that the backgrounds of the ARPES spectra reported in refs^21,22^ are low as those shown in Fig. 3c,d, we conclude the different number of HOMO band to be the reason of the mismatch. As mentioned above, the presence of only one HOMO band is assumed in refs^20–22^ though the existence of two HOMO-derived bands is obvious from Fig. 3c,d. By analyzing the present ARPES results as in refs^20–22^, i.e., by following the data point with maximum intensity in the HOMO region, the “artificial HOMO-derived band” of the present study (dashed line in Fig. 4) gives a dispersion width of approximately 370 meV along the Γ-Y direction, i.e., a value comparable with those reported in these literatures. We therefore conclude that the reason of the contradiction between the present dispersion width and those in refs^20–22^ is the underestimation of the number of HOMO-derived band, which comes from ignoring the HOMO-derived component with lower intensity.

**Figure 5:**
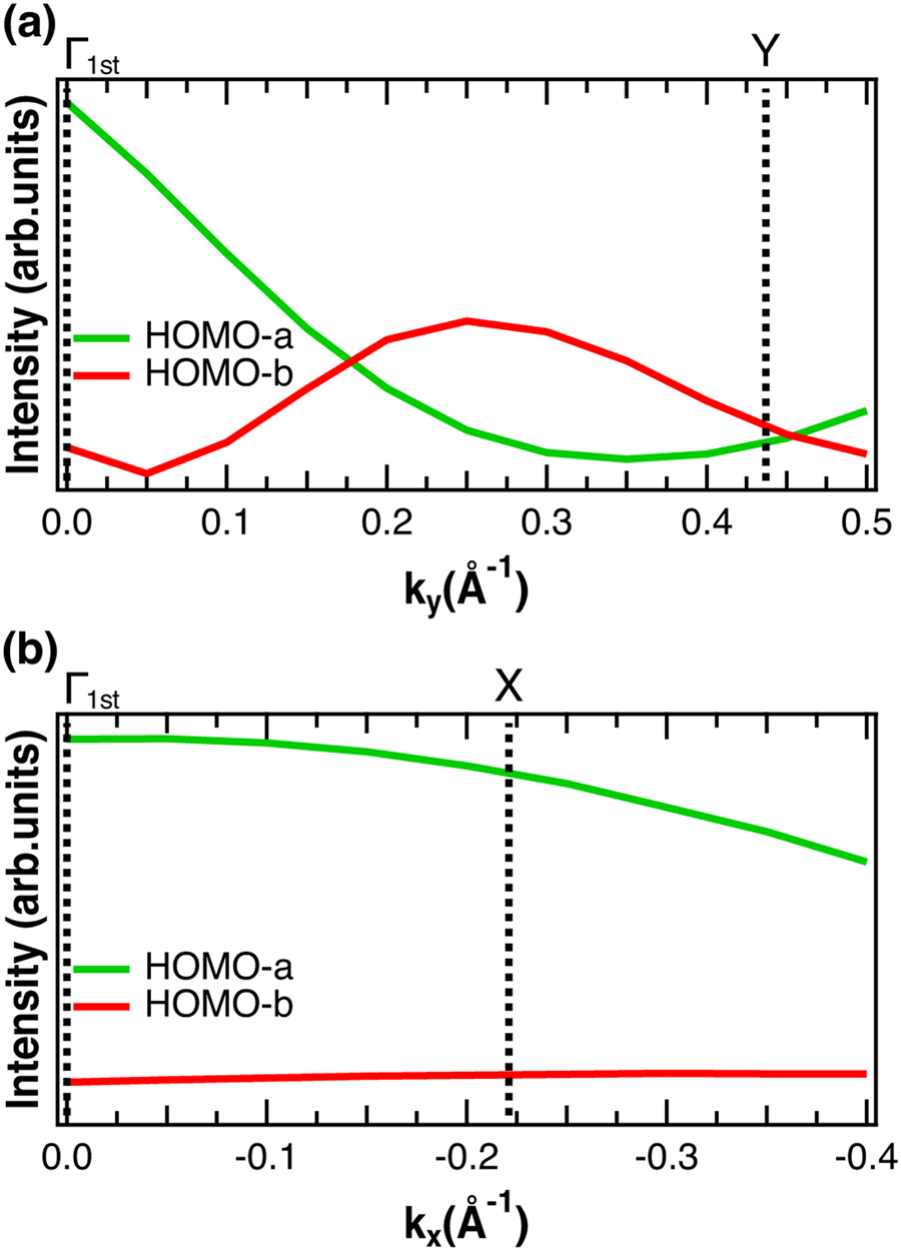
Photoelectron diffraction effect induced intensity variation of the HOMO-a and HOMO-b bands obtained by theoretical calculation. (**a,b)** Show the results along the Γ-Y and Γ-X directions, respectively. The intensities are normalized at the intensity of HOMO-a at k_//_ = 0 Å^−1^.

The reason of missing the second HOMO-derived band would result from the variation in intensity of the two HOMO-derived components. The size of the circles in Fig. 4 corresponds to the intensity of the HOMO-a and HOMO-b components obtained experimentally along the Γ-Y and Γ-X directions. This figure clearly shows that the intensity of HOMO-a is always larger than that of HOMO-b along the Γ-X direction, while the relative intensity of HOMO-a and HOMO-b is reversed at the center of the BZ along the Γ-Y direction. These relative intensity variations of HOMO-a and HOMO-b are well explained by the PED simulation shown in Fig. 5. Both the HOMO-a and HOMO-b show negligible PED effect along the Γ-X direction, whereas PED effect leads to a reverse of the relative intensity of the two HOMO-derived bands at around 0.2 A^−1^ along the Γ-Y direction. This qualitative agreement between the experimental and PED simulation results indicates that photoelectron diffraction would be the reason of the different intensity variation behavior along the two directions, and thus the origin of underestimating the number of HOMO-derived bands in the former ARPES studies.

Considering the conditions of the present experimental study and the former theoretical ones, the difference in sample temperature would be the key to understand the discrepancy in dispersion width. All theoretical calculations were performed at a sample temperature of 0 K, i.e., all calculated band structures are based on frozen molecules, while the present ARPES measurement was done at room temperature, a temperature where molecules are vibrating violently. Since vibrations change the orbital overlap of adjacent molecule and thus the band dispersion, we conclude that the smaller dispersion width obtained in the present study result from the finite temperature of the sample that was ruled out in the theoretical calculations. (The molecular vibration can also explain the disagreement in the quantitative intensity variation discussed above as shown in Supplementary Fig. S3).

Finally, we discuss the carrier transport mechanism of a rubrene SC at room temperature. The hole effective mass (${m}_{h}^{\ast }$) of the HOMO bands, which can be obtained from the second derivative of the dispersion as $1/{m}_{h}^{\ast }=1/{\hslash }^{2}\cdot {d}^{2}{E}_{B}/d{k}_{||}^{2}$, is given by ${m}_{h}^{\ast }={\hslash }^{2}/2t{b}^{2}$ around the Γ point. This leads to an estimation of ${m}_{h}^{\ast }=3.7{m}_{0}$ along the Γ-Y direction in the present study, and by using the relation ${\mu }_{h} > 20({m}_{0}/{m}_{h}^{\ast })\times (300/T)$, where *μ*_*h*_ is the hole mobility and is valid in case of $W > {k}_{B}T$ ^34^, the *μ*_*h*_ is estimated to be ${\mu }_{h} > 5.4$ cm^2^/Vs along the *b* direction of the unit cell. This value is much smaller than those obtained by transport measurements, and therefore indicates that the HOMO bands are not the main channel of carrier transport though band-like mechanism is suggested in the former transport studies^31,32^. Since the dispersions of HOMO bands at room temperature are different from those calculated at 0 K due to thermal effect, molecular vibrations mediated carrier transport such as hopping mechanism enhanced by molecular vibrations can be the origin of the high carrier mobility.

## Conclusion

In the present study, we demonstrated that a band-like mechanism observed in transport measurements does not mean that the MO bands are the main channel for carrier transport in case of organic SCs. Taking the difference between the dispersions of the HOMO-derived bands obtained experimentally at room temperature and those obtained theoretically into account, we propose that a more complex picture such as molecular vibrations mediated carrier transport would be the origin of the high carrier mobility in rubrene SC. Thus, this work will open a new avenue to realize organic semiconductor devices with high carrier mobility.

## Methods

### Sample preparation

Rubrene single crystal was grown by physical vapor transport method. We have repeated sublimation for 5 times to purify the rubrene powder used as the source material. The powder were placed in the hot zone of the tube furnace (~573 K), and rubrene single crystal were grown on the wall of a glass tube where the temperature was kept at the crystallization region (~513 K) in a ~30 SCCM stream of highly purified Ar gas (>~99.999%).

### Transport measurement

We have used a well structure of polydimethylsiloxan (PDMS) elastomer as the substrate and used an air-gap gate insulator with thickness of 25 μm as gap layer. Rubrene SC was electrostatically attached on the Au electrodes. All the measurements were done in air at room temperature with an Agilent Technology B1500A semiconductor parameter analyzer.

### ARPES measurements

All ARPES measurements were performed in UHV (<1 × 10^−10^ mbar) at room temperature. A SPECS GmbH PHOIBOS 100 photoelectron analyzer was used at the beamline I4 at MAX IV, Sweden and a VG-SCIENTA SES2002 analyzer was used at the APE beamline at Elettra, Italy, with a linearly polarized synchrotron radiation light (*hν* = 30, 40 eV). The energy resolution was below 25 meV and the momentum resolution was below 1% of the Brillouin zone. Rubrene SC is electrostatically laminated on top of a Si substrate, whose SiO_2_ layers were removed by HF etching, and Ag paste is used to cover the edge of SC to ground the sample.

### Theoretical calculation

The photoemission intensity was calculated based on the Fermi’s golden rule, and a boundary condition where the wave function is formed by fully interacting incoming waves and a plane wave which describes electrons into the detector. The initial states were obtained by Hartree-Fock calculation using Gaussian 09^35^, and a calculated ground state electronic structure for two rubrene molecules with a STO-3G basis set. Regarding the final states, we apply multiple scattering theory^36,37^, in which the whole space is divided into small cells. As an approach for partition, we employed the muffin-tin (MT) approximation where the whole space is divided into two type of regions; spherically symmetric potential regions (MT spheres) located at atoms, and the remaining “interstitial” regions where the potential is constant. The scattering potential is constructed from a self-consistent field charge density obtained by Gaussian 09, and the partial derivatives of the initial state orbitals were expanded by a series of spherical harmonics in each MT spheres^38^. The alignment of the light, photoelectron, and molecules used in the PED simulation is shown in the Supplementary Fig. S4.

## Supplementary information

Supplementary information

## Data Availability

All data needed to evaluate the conclusion of this paper are presented in the paper and/or the supplementary materials. Additional data are available from the corresponding author upon reasonable request.
