# Supplementary material for: The actual electronic band structure of a rubrene single crystal
Source: Sci Rep. 2019 Jul 4;9:9645. doi: 10.1038/s41598-019-46080-4 (PMC6609628; doi:10.1038/s41598-019-46080-4)
Supplement: Supplementary file 1 — Supplementary information [file 41598_2019_46080_MOESM1_ESM.pdf]

Supplementary information for "The actual electronic band structure of a rubrene single crystal"

Jun Nitta[1*], Kazumoto Miwa[2], Naoki Komiya[1], Emilia Annese[3†], Jun Fujii[4], Shimpei Ono[2], and Kazuyuki Sakamoto[1,5,6,7**]

[1] Department of Nanomaterials Science, Chiba University, Chiba 263-8522, Japan

[2] Central Research Institute of Electric Power Industry, Yokosuka 240-0196, Japan

[3] Elettra-Sincrotrone Trieste S.C.p.A, I-34149 Trieste, Italy

[4] Istituto Officina del Materiali (IOM)-CNR, Laboratorio TASC, I-34149 Trieste, Italy

[5] Department of Materials Science, Chiba University, Chiba 263-8522, Japan

[6] Molecular Chirality Research Center, Chiba University, Chiba 263-8522, Japan

[7] Department of Material and Life Science, Osaka University, Osaka 565-0871, Japan

[†]Present address: Centro Brasileiro de Pesquisas Físicas (CBPF), Rua Dr. Xavier Sigaud 150, Urca, Rio de Janeiro 22290-180, Brazil.

* jn.nitta25@gmail.com

** kazuyuki_sakamoto@ap.eng.osaka-u.ac.jp

Surface sensitivity.

ARPES is a surface sensitive method due to the limited mean free path of electrons traveling inside materials. In case of using a photon energy of 30 eV, the energy of photoelectron emitted from the HOMO band is approximately 24.5 eV, which leads to a mean free path of approximately 1 nm. However, this mean free path does not imply that bulk bands cannot be observed in ARPES. In fact, bulk valence bands and/or the projection of bulk valence bands were observed in former studies performed on inorganic materials. Thus, if the band has spatially different structure in the surface normal direction, we should observe bands from both surface and bulk, or at least traces of different bands in the ARPES images and spectra. However, since such trace is not observed in Figs. 3 and 4, we are convinced that there is no spatially different band structure and that the observed bands are those of bulk rubrene.

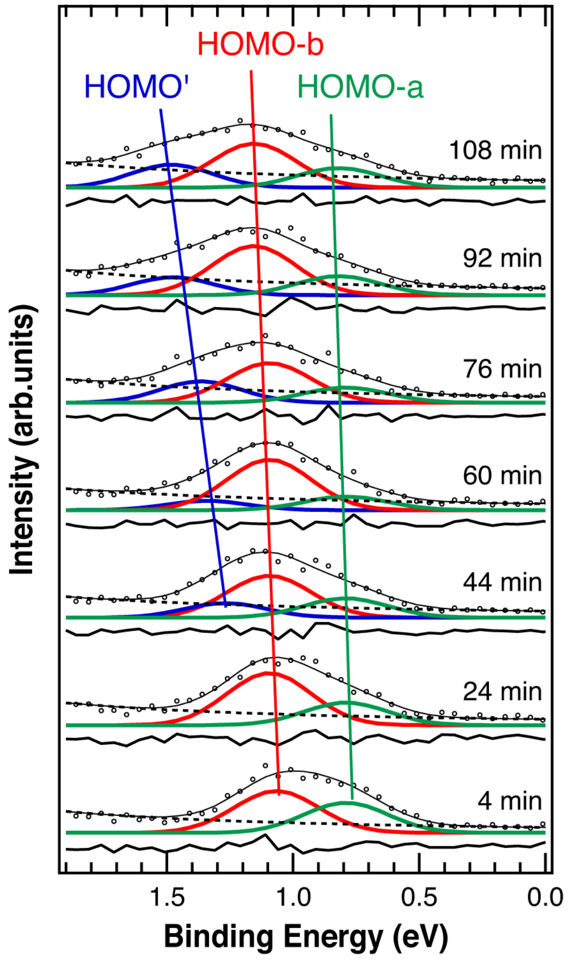

Figure S1. Time-dependent ARPES spectra at the HOMO region at k_x=k_x=0 Å^{-1} obtained with $h\nu$=40 eV from a low quality rubrene SC. The open circles are the experimental data, and the solid lines overlapping these circles are the fitting results obtained by the components (HOMO-a, HOMO-b, HOMO') indicated below each spectrum. The solid line at the bottom of each spectrum is the residue between the experimental data and the fitting result. The time indicated at the right of each spectrum is the time after starting the measurement. The lowest spectrum can be fitted using the two components observed in high quality sample, HOMO-a and HOMO-b, but the intensity of the HOMO-a is smaller than that of HOMO-b and the background intensity at the left side of the peak is higher than that of the right side. By continuously irradiating light on the sample, the two components shift to higher binding energy with a reduce in the relative intensity of HOMO-a to HOMO-b (reduction in $I_{HOMO\text{-}a}/I_{HOMO\text{-}b}$), and a

new component (HOMO’) appears at the higher binding energy side of HOMO-b. These results, which were not observed on high-quality samples, show good agreements with the features observed on a photo-induced damaged sample reported in [Machida, S.-i. *et al.*, *Phys. Rev. Lett.* **104**, 156401 (2010)].

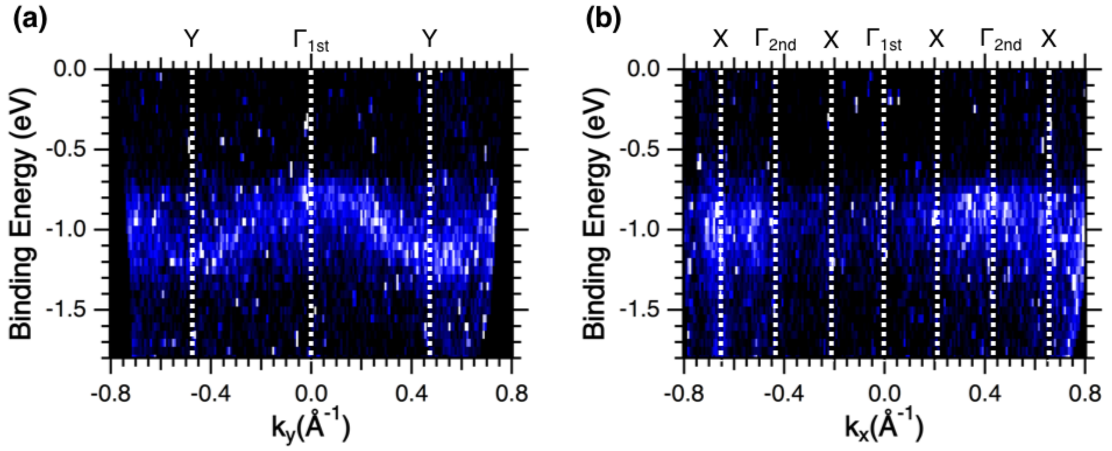

Figure S2. ARPES images of the HOMO region obtained at $h\nu$=40 eV. **a** shows the dispersion along the Γ-Y direction and **b** is the dispersion along Γ-X. Brighter area corresponds to higher photoelectron intensity. These two images were recorded at every 0.07°, the same interval at the image shown in Fig. 4**b**, but different from that shown in Fig. 4**a** that was recorded at every 0.5°. The dispersion widths are approximately 370 meV in **a** and negligible in **b**. These widths agree well with those obtained using $h\nu$=30 eV. The lower quality of the data compared to those obtained at $h\nu$=30 eV (Figs. 3 and 4) would be due to the lower cross section at this photon energy (note that the quality of data obtained at $h\nu$=40 eV was lower than that at $h\nu$=30 eV in Nakayama, Y. *et al.*, *Appl. Phys. Express.* **5**, 111601 (2012) as well).

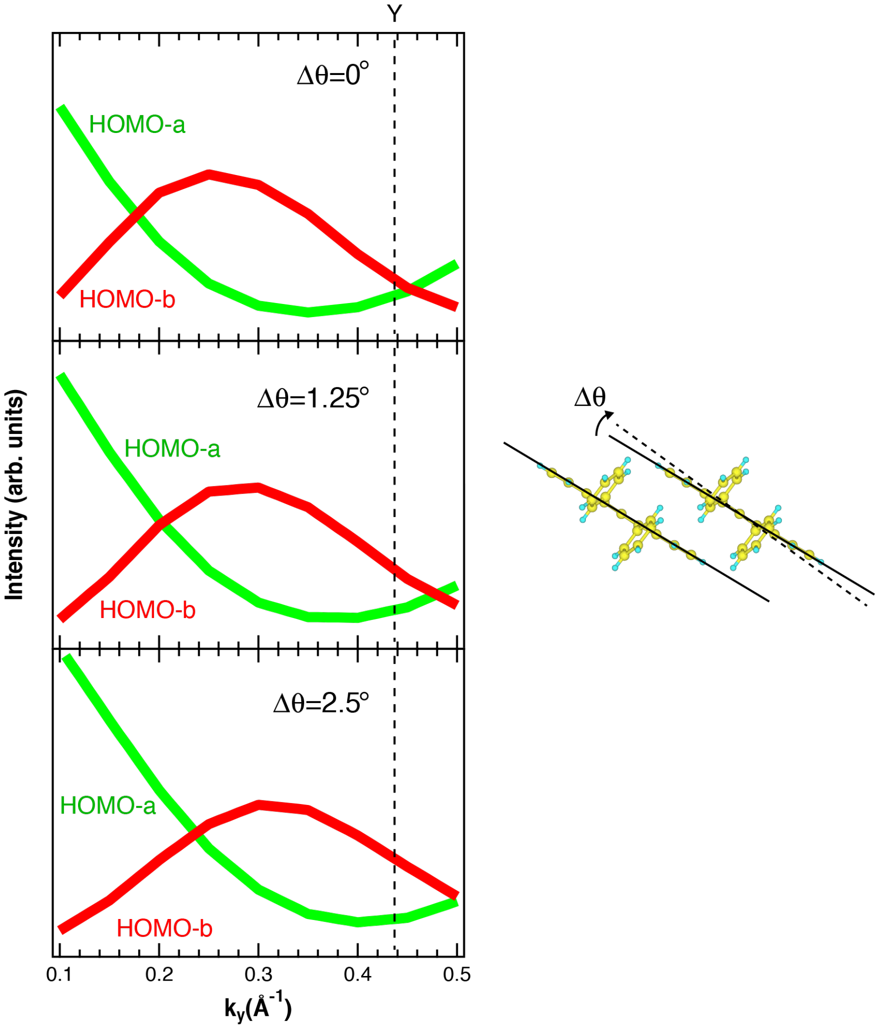

Figure S3. Effect of the molecular vibration to the intensity of the HOMO-a and HOMO-b when considering a simple vibrational mode. By fixing one molecule (the left one of the schematic drawing) and rotating the other in the anticlockwise direction, the intensity difference at the Y point becomes larger. ($\Delta\theta$=0° means that rubrene molecules are parallel.) This result support that molecular vibration would be the origin of the disagreement in the quantitative intensity variation of HOMO-a and HOMO-b.

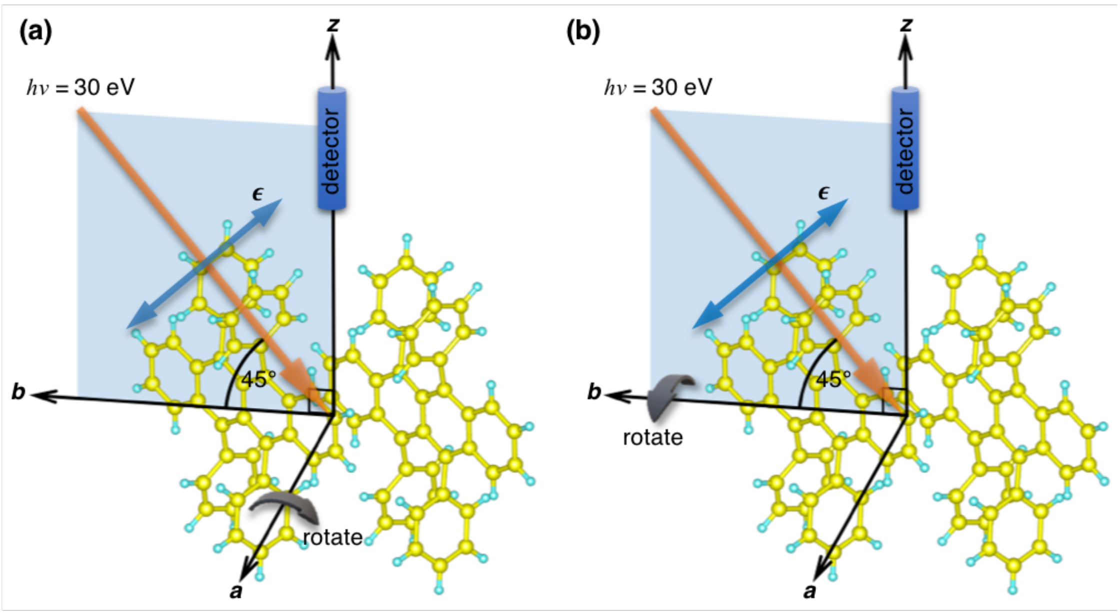

Figure S4. Alignment of the light, photoelectron, molecules used in the PED simulation. **a,b** are the alignment for the simulation along Γ-Y and Γ-X, respectively.
